# A Novel Paradigm Between Leukocytosis, G-CSF Secretion, Neutrophil-to-Lymphocyte Ratio, Myeloid-Derived Suppressor Cells, and Prognosis in Non-small Cell Lung Cancer

**DOI:** 10.3389/fonc.2019.00295

**Published:** 2019-04-26

**Authors:** Montreh Tavakkoli, Cy R. Wilkins, Jodi V. Mones, Michael J. Mauro

**Affiliations:** ^1^Department of Internal Medicine, New York-Presbyterian Hospital/Weill Cornell Medical Center, New York, NY, United States; ^2^Department of Hematology, Memorial Sloan Kettering Cancer Center, New York, NY, United States; ^3^Department of Hematology Oncology, Memorial Sloan Kettering Cancer Center, New York, NY, United States

**Keywords:** granulocyte-colony stimulating factor (G-CSF), myeloid derived suppressor cell (MDSC), biomarker (development), non-small cell lung cancer, leukocytosis, paraneoplastic leukocytosis, NLR, neutrophil to lymphocyte ratio (NLR)

## Abstract

Leukocytosis is a common feature of malignancies. While controversial, there appears to be an association between the degree of tumor-related leukocytosis and prognosis. In this paper, we provide evidence supporting an untapped clinical paradigm linking G-CSF secretion to the induction of leukocytosis and expansion of myeloid-derived suppressor cells, providing an explanation for the association between leukocytosis, elevated neutrophil-to-lymphocyte ratios and prognosis in non-small cell lung cancer. Clinically validating this mechanism may identify MDSCs and G-CSF as dynamic markers of early disease progression and therapeutic response, and shed light onto novel therapeutic avenues for the treatment of patients with non-small cell lung cancer.

## Introduction

Leukemoid reactions are defined by the presence of white counts of >50 k/μl (normal range 4–11 k/μl), and are considered paraneoplastic when other causes have been excluded. Paraneoplastic leukemoid reactions (PLRs) are rare phenomena that are more commonly reported in case reports, whereas milder forms of leukocytosis are commonly observed in solid tumors (1). Studies have shown that among patients with newly diagnosed lung cancer, 14.5% have leukocytosis, and ~20% of those with leukocytosis have a solid malignancy (2). While PLR is distinguished from milder forms of leukocytosis in the scientific literature, the two are likely part of a continuum defined by the degree of cytokine production, and are thus herein referred to as “tumor-related leukocytosis.” Tumor-related leukocytosis is associated with poor patient outcomes (1, 2). However, studies evaluating the mechanisms underlying this association are limited. We performed an extensive review of the literature and propose a novel mechanism by which leukocytosis and G-CSF production are likely linked to neutrophil-to-lymphocyte ratios (NLR), tumor progression, metastasis, and thus poorer outcomes via MDSCs in patients with non-small cell lung cancer (NSCLC).

## Leukocytosis and Prognosis

**Figure 1:**
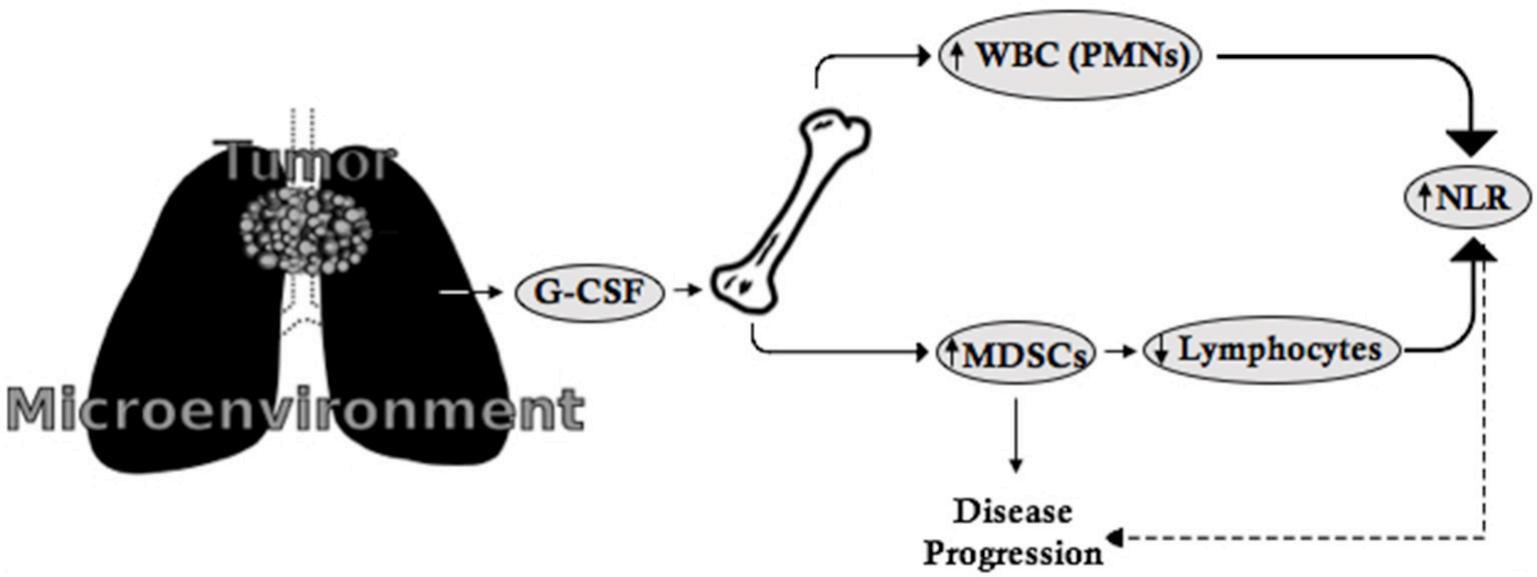
The G-CSF-MDSC-NLR paradigm. G-CSF is secreted by tumors or the tumor microenvironment and leads to the proliferation of leukocytes (including neutrophils) as well as MDSCs from the bone marrow. MDSCs suppress lymphocyte expansion and proliferation. As a consequence, the increased number of neutrophils and suppression of lymphocytes leads to increased NLR, which is indirectly related to disease progression, while MDSCs directly contribute to disease progression and metastasis.

In clinical practice, little attention is paid to understanding the underlying etiology of leukocytosis in cancer patients once infectious processes and/or underlying hematologic disease have been ruled out. However, the mechanisms that mediate tumor-related leukocytosis appear to play a significant role in the underlying pathophysiology of lung cancer progression and prognosis. The scientific literature suggests that (a) tumor-related leukocytosis may be driven by increased baseline hematopoietic growth factor (e.g., G-CSF) secretion by tumors or tumor microenvironments; (b) G-CSF secretion fosters myelopoiesis and the expansion of MDSCs, which may represent a subset of cells that increase with worsening leukocytosis; (c) MDSCs, which appear to be detected on routine CBCs as neutrophils and restrict T cell proliferation and expansion may explain the elevated NLR observed in NSCLC; and (d) MDSCs, which play a role in tumor progression and metastasis, may therefore provide a potential explanation for the association between G-CSF, tumor-related leukocytosis, NLR, and poor patient outcomes (3–5) (Figure 1).

Leukocytosis has been historically associated with a poor prognosis in patients with solid tumors. In a study of 227 patients with newly diagnosed lung cancer, the median survival was 4.6 vs. 20.8 months in those with elevated and normal white counts (*p* < 0.001) (2). In a pooled analysis of the North Central Cancer Treatment Group Trials, 1,053 patients with advanced NSCLC were evaluated and leukocytosis was found to be a negative prognostic factor for overall survival (OS) (hazard ratio [HR] for death 1.43, *p* < 0.0001, 95% CI 1.22–1.67) and time to progression of disease (HR 1.37, *p* < 0.0001, 95% CI 1.17–1.62). Six-month and 1-year OSs were 30 vs. 43% and 6 vs. 14% for leukocytosis vs. the absence of leukocytosis (6). Furthermore, among patients with white counts of >40 k/μl, 78% die or are discharged to hospice within 12 weeks of their initial profound tumor-related leukocytosis (1). Despite these promising results, the use of leukocytosis as a prognostic marker has been controversial in lung cancer. This is likely due to leukocytosis being an indirect measure of G-CSF, which appears to play a role in tumor progression.

## G-CSF, Leukocytosis, and Prognosis

G-CSF levels are elevated in patients with NSCLC relative to healthy individuals (103.2 vs. 24.0 pg/mL, *p* < 0.001) and higher levels appear to be associated with a poorer prognosis (7). While in one study, Katsumata et al. found no significant difference between the G-CSF levels in healthy volunteers relative to individuals with lung cancer, this discrepancy is likely due to the low power and inclusion of lesser patients with advanced disease in this study relative to the former (87 vs. 69% stage III/IV disease) (7, 8). In support of this, preclinical *in vivo* models have revealed that the administration of G-CSF to mice injected with non-metastatic cell lines leads to metastatic behavior and inoculation with metastatic cell lines leads to an increased number of lung metastases (3, 8). In addition, Katsumata et al. found more frequent metastases to the adrenal glands in patients with high G-CSF levels relative to low G-CSF (67 vs. 7%, *p* < 0.001), suggesting that G-CSF plays an oncogenic role in tumor growth and metastasis (8).

Furthermore, in a retrospective analysis of 89 patients with solid tumors including NSCLC, Stathopoulos et al. (9) found that 60% of patients had G-CSF levels <100 pg/ml and 40% had levels >100 pg/ml, respectively, and that those with G-CSF levels <100 pg/ml, >100 pg/ml, >200 pg/ml, and >1,000 pg/ml had white counts of 4–10 k/μl, 8–12 k/μl, 10–20 k/μl, and 22–240 k/μl and median survivals of 12, 9, 7 months, and 1 week (9). While no statistical analyses were performed, this study suggests a link between G-CSF level, white count, and prognosis. Importantly, overlapping white counts within distinct prognostic categories based on G-CSF level suggest that leukocytosis may be limited in clinical utility as a discriminator of prognosis. Thomson et al. came to a similar conclusion in a study of 44 patients with lung cancer vs. 75 healthy adults (10). Thus, G-CSF level may serve as a better surrogate for disease progression and prognosis than leukocytosis. However, prospective studies evaluating G-CSF levels and its association with tumor progression and metastasis in humans are necessary to confirm this.

## G-CSF, MDSC's, and Prognosis

G-CSF likely mediates its effects by inducing the proliferation and mobilization of MDSCs—pathologically activated myeloid precursors. There are two major subtypes of MDSCs, polymorphonuclear and mononuclear MDSCs (PMN-MDSCs and M-MDSCs), that resemble granulocytes and monocytes in morphology (11). MDSCs induce changes in cellular function and the tumor microenvironment that promote tumor progression and metastasis, but are not involved in the initial phase of tumor development (11). Such changes include the stimulation of mesenchymal to epithelial transition, angiogenesis, vascular remodeling as well as immune evasion via depleting L-arginine and L-tryptophan in the tumor microenvironment, leading to T cell cycle arrest and anergy, expanding Tregulatory cells, impairing the functioning of NK cells, and directly suppressing CD8+ T cells (4, 5, 12, 13). Patients with NSCLC have significantly higher MDSCs relative to healthy patients, and the frequency of MDSCs correlate with poor cancer stage, metastatic burden, response to chemotherapy and progression-free survival (PFS) (3- vs. 9-month PFS, HR 0.30, *p* = 0.03) (4, 5, 14–18).

In preclinical models, tumor-secreted G-CSF has been shown to expand and mobilize MDSCs from the bone marrow and to facilitate MDSC homing into distant organs, with MDSCs creating a pro-tumorigenic microenvironment that supports tumor extravasation and metastasis (3). Further, Kowanetz et al. found that pre-treating mice with G-CSF enhances the metastatic properties of tumors and promotes the invasive behavior of non-metastatic tumors, while targeting MDSCs with antibodies significantly reduces G-CSF-induced metastasis (3). Similarly, a study by Shojaei et al. (19) revealed the preferential expression of G-CSF in refractory tumors and that treatment with anti-G-CSF antibody significantly reduces circulating and tumor-associated MDSCs, delays growth and inhibits tumor angiogenesis via Bv8 blockade (19). Thus, G-CSF is likely part of a pro-oncogenic program that mediates angiogenesis, tumor growth, and metastasis through the effects of MDSCs.

This raises the question as to whether G-CSF administration may impact survival. There is no current consensus for a benefit in survival with the use of G/GM-CSF in NSCLC (20). In a randomized control study of 52 patients with NSCLC who were treated with chemotherapy and GM-CSF vs. placebo, for instance, median survival was 10 months in both arms (21). However, there are multiple reasons for which G-CSF administration may not worsen prognosis. (1) G-CSF enables greater dose intensification and prevents dose-reductions in chemotherapy, thus allowing patients to receive greater cytotoxic regimens. For instance, paclitaxel 120 mg/m^2^ results in dose-limiting neutropenia, while G-CSF allows paclitaxel dose-escalation to 250 mg/m^2^ (22). Furthermore, chemotherapy is dose-reduced by ≥15% in most patients who do not receive G-CSF (23). While controversial, dose intensification may lead to a survival benefit (24). (2) Persistent G-CSF stimulation may be necessary to induce stable MDSC expansion/proliferation, and thus short-courses of G-CSF may not impact outcomes. Patients receive only 2.6 doses of G-CSF, respectively, according to a study of 180 patients treated at the Cleveland Clinic (25). Lastly, (3) G-CSF reduces infectious complications of chemotherapy and prolonged hospitalization that are associated with higher mortality (23, 26, 27).

## NLR, MDSC's, and Response to Surgical Resection/Chemotherapy

Tumor microenvironments play a significant role in cancer pathogenesis, and biomarkers assessing patient responses to therapy are lacking in clinical practice. Thus, the NLR, a surrogate for tumor-associated inflammation, has been evaluated as a prognostic marker in NSCLC. In a study of 88 patients with stage IV NSCLC, higher baseline NLR correlated with poorer disease outcomes (OS: NLR ≤ 4 vs. NLR >4: 21.4 and 6.8 months, 95% CI 0.23–0.88, *p* = 0.019) (28). In a meta-analysis of 7 articles involving 738 patients with melanoma, genitourinary malignancies, and NSCLC, high NLR was associated with worse OS and PFS across all immunotherapies (OS: HR 1.92, 95% CI 1.29–2.87, *p* = 0.01 and PFS: HR 1.66, 95% CI 1.38–2.01, *p* < 0.00001) (29). Furthermore, in a meta-analysis of 14 studies involving 3,656 patients with NSCLC, elevated pretreatment NLR continued to predict poorer OS and PFS (OS: HR 1.70, 95% CI 1.39–2.09, *p* < 0.001 and PFS: HR 1.63, 95% CI 1.27–2.09, *p* < 0.001) (30). The mechanisms by which elevated NLR is associated with a poor prognosis is not well understood, however, MDSCs may provide an explanation for this phenomenon.

Rather than broadly reflecting tumor-associated inflammation, NLR likely represents MDSC frequency and activity. Specifically, G-CSF induces myelopoiesis that leads to neutrophilia and increases the frequency of MDSCs, while MDSCs inhibit T cell proliferation and expansion and thus limit the number of circulating lymphocytes. Taken together, these processes elevate the numerator and reduce the denominator of NLR to increase the NLR. In support, both elevated NLR and MDSCs are associated with a poor prognosis. In addition, a study of 58 patients with stage I-IV NSCLC revealed that NSCLC is associated with higher neutrophil counts (5,425 vs. 3,854 cells/ul, *p* < 0.001), lower naïve CD4+ (42% CD4+ vs. 28%, *p* < 0.001) and thus higher NLRs (3.1 vs. 1.95, *p* < 0.001), as well as higher frequencies of M-MDSCs (6.5% vs. 2.61% cells/ul, *p* < 0.001) (18). Unfortunately, no study has assessed the relationship between MDSCs and NLR, however, this study drew positive correlations between MDSCs and neutrophil counts (*r* = 0.434, *p* < 0.001) and negative correlations between MDSCs and naïve CD4+ T cells (*r* = −0.248, *p* = 0.01). Further, higher MDSC frequency is associated with lower OS (HR 8.301, *p* = 0.046), as are lower numbers of naïve CD4+ T cells (*p* = 0.037) and CD8+ T cells (*p* = 0.041) (18). In line with these findings, others have observed lower mean absolute neutrophil counts in patients with objective responses (4.2 vs. 6.6 cells/μl, *p* = 0.017) and better disease control (4.9 vs. 7.9 cells/μl, *p* < 0.001) relative to those with stable or progressive disease and inverse correlations between MDSCs and CD8+ T cells (*r*^2^ = −0.3141, *p* = 0.0297) and CD4+ T cells (*r*^2^ = −0.3, *p* = 0.006) (15, 17, 28).

While NLR appears to be valuable for prognostication, MDSCs may serve as a more accurate marker of prognosis given that NLR is potentially an indirect measure of MDSCs. However, no single study has performed a head-to-head comparison to assess this. There is, however, direct evidence that MDSC monitoring may be effective for the evaluation of therapeutic response. Reductions in MDSCs are observed in patients with early-stage NSCLC following tumor resection (34.12% pre- vs. 7.66% post-operatively, *p* = 0.0391) (15). In patients treated with platinum-based chemotherapy, elevated M-MDSCs vary with respect to response to treatment, with higher numbers in progressive disease relative to stable disease and partial remission (27.2, 20.6, and 14.7%, *p* < 0.001) (5). Others have come to similar conclusions and have found that higher M-MDSCs are an independent prognostic factor for decreased PFS and OS (PFS: HR 2.41, 95% CI 1.37–4.24, *p* = 0.002 and OS: HR 2.35, 95% CI 1.25–4.41, *p* = 0.008) in patients treated with chemotherapy, and are inversely correlated with PFS in patients who undergo surgical resection (95 vs. 50%, respectively, *p* = 0.014–0.019) (15–17). Thus, MDSC frequency may be useful in detecting disease recurrence in those who undergo surgical resection and/or receive standard chemotherapy.

## NLR, MDSC's, and Response to Immunotherapy

PD-1 and PD-L1 inhibitors have been approved for the treatment of NSCLC. While pembrolizumab monotherapy has been approved for patients expressing ≥50% PD-L1, pembrolizumab combination therapy, atezolizumab, and nivolumab have also been approved for advanced NSCLC irrespective of PD-L1 expression (31–34). Only 44.8% of patients with ≥50% PD-L1 expression respond to pembrolizumab, while 45.3% of those with <1% PD-L1 expression receiving nivolumab and ipilimumab respond to therapy, suggesting that PD-L1 is far from an ideal biomarker for response to immunotherapy (31, 35).

NLR has been investigated as a predictor of response to immunotherapy and is promising in patients with advanced NSCLC. In 30 patients with NSCLC who were treated with nivolumab, for instance, the median PFS for NLR <5 and NLR ≥5 at 4 weeks was 95 days and 10 days (HR 5.995, 95% CI 1.225–29.35, *p* = 0.0271), indicating that NLR ≥5 is associated with poorer responses at 4 weeks post-nivolumab therapy (36). In a retrospective analysis of 54 patients with NSCLC who received nivolumab or pembrolizumab, no patients with 6-week post-treatment NLR ≥5 achieved PR (regardless of PD-L1 expression), whereas 42% of patients with post-treatment NLR <5 achieved PR (*p* = 0.011), suggesting that NLR ≥5 may identify non-responders at 6 weeks post-nivolumab and -pembrolizumab therapy. The median PFS and OS of 6-week post-treatment NLR >5 vs. NLR ≥5 were 1.3 and 6.1 months (*p* < 0.001) and 2.1 and 14.0 months (*p* < 0.001) (37). In a multivariate analysis, high post-treatment NLR at 6 weeks was found to be an independent prognostic factor for shorter PFS (HR 15.09, 95% CI 4.55–50.06, *p* < 0.001) and OS (HR 3.82, 95% CI 1.59–9.17, *p* = 0.003) (37). To provide even further evidence for the clinical utility of NLR in patients treated with PD-1 axis inhibitors, Park et al. (38) created a prognostic calculator using baseline NLR and changes in NLR to categorize 159 patients with advanced NSCLC into good, intermediate, and poor risk groups with respect to response to nivolumab. The model was found to be a robust predictor of PFS and OS, with 12-month OS for good, intermediate, poor being 87.8, 64.1, and 28.2%, with a poor group odds ratio of 9.59 (95% CI 3.8–26.9, *p* < 0.0001), and was confirmed in an independent cohort (OS: 15.9, 12.4, and 4 months, *p* = 0.001, with poor risk group HR 2.7, CI: 1.2–6.1, *p* = 0.013) (38, 39).

Data is limited with respect to MDSCs and prognosis in response to immunotherapy, hence our particular interest in this topic. In a pilot study of 34 patients with NSCLC, however, Kim et al. (40) prospectively enrolled patients who had failed platinum-based chemotherapy and found that at a cut off of 0.39, patients with Treg/PMN-MDSC ratios of ≥0.39 had significantly longer median PFSs relative to those with Treg/PMN-MDSCs <0.39 (103 vs. 35 days, *p* = 0.0079). A validation cohort involving 29 patients further supported these findings (Treg/PMN-MDSCs ≥0.39 vs. <0.39: PFS not reached vs. 35 days, *p* = 0.0017). To elucidate the mechanism involved, the authors analyzed 40 cytokines and chemokines following the first nivolumab infusion (excluding G-CSF) and found that CXCL2, CCL23, and CX3L1, which have been reported to promote MDSC recruitment, and HMGB1, involved in MDSC differentiation, were elevated (40).

## MDSC's as Dynamic Markers of Therapeutic Response and Early Disease Relapse

While some studies suggest that NLR and MDSC frequency at baseline could be used to predict outcomes, changes in NLR and MDSC frequency are likely to be better predictors of early disease relapse. In a study of 30 patients who received nivolumab, for instance, the median PFS for NLR <5 and NLR ≥5 at baseline, 2 and 4 weeks were 82 vs. 40 days (HR 1.226, 95% CI 0.3–4.8, *p* = 0.77), 67 vs. 109 days (HR 0.6647, 95% CI 0.1837–2.406, *p* = 0.5537) and 95 vs. 10 days (HR 5.995, 95% CI 1.225–29.35, *p* = 0.0271) (36). While the differences were significant at 4 weeks, there was no statistically significant difference at baseline and 2-weeks post-treatment. Similarly, 6-week post-treatment NLR in 54 patients with advanced NSCLC who received nivolumab or pembrolizumab was significantly associated with PFS and OS, however, baseline NLR was not associated with outcomes (37). These studies may have been underpowered, particularly given trends toward a difference. In contrast, they may suggest that dynamic changes in NLR better represent a patients' disease status. Evidence for this stems from a small study of 15 patients with advanced NSCLC treated with nivolumab, where NLR increased overtime in non-responders and decreased in responders (*p* = 0.045). In addition, there was an inverse relationship between drug response and 6-week NLR (*p* = 0.048) (41). In another study, trending NLR among patients with stage IV NSCLC who had received an PD-1 axis inhibitor helped identify patients with objective responses (36.4 vs. 13.6%, *p* = 0.025) and better disease control (29.5 vs. 6.8%, *p* = 0.011) in response to treatment (28). NLR decreased by 1.5x in patients with objective responses relative to those with stable or progressive disease who had increasing NLR by 2.4x at 8-weeks post-treatment (*p* = 0.020) (28). Data is limited with respect to MDSCs, however, among 46 treatment-naïve patients who received platinum-based chemotherapy ± bevacizumab, those with disease progression were also found to have significantly increased CD15+ M-MDSCs from baseline (*p* = 0.03) (42). Collectively, changes in NLR and MDSCs may serve as better measures of outcomes than absolute NLR or MDSC frequency at any single time point.

## Conclusions

Collectively, tumor-related leukocytosis has been largely ignored and understanding the mechanisms underlying this process may lead to the discovery of novel biomarkers and targets for the prognostication and management of patients with NSCLC. Large prospective studies are necessary to elicit whether there is a direct correlation between G-CSF, leukocytosis, MDSCs, NLR and prognosis in NSCLC, and to assess whether dynamic changes in G-CSF and MDSCs could be used to monitor disease progression and response to therapy. Further elucidating the contribution of G-CSF and MDSCs to malignant pathogenesis may also prove critical to understanding the mechanism by which NLR correlates with prognosis and to the development of novel therapeutic strategies targeting the GCSF- MDSC pathway as anti-angiogenic, anti-metastatic and immunomodulatory therapies.

## Author Contributions

MT wrote the manuscript. CRW, JVM and MJM revised the article.

### Conflict of Interest Statement

The authors declare that the research was conducted in the absence of any commercial or financial relationships that could be construed as a potential conflict of interest.
